# The Role of AMPK in the Regulation of Skeletal Muscle Size, Hypertrophy, and Regeneration

**DOI:** 10.3390/ijms19103125

**Published:** 2018-10-11

**Authors:** David M. Thomson

**Affiliations:** Department of Physiology & Developmental Biology, Brigham Young University, Provo, UT 84602, USA; david_thomson@byu.edu; Tel.: +1-801-422-8709

**Keywords:** AMPK, LKB1, autophagy, proteasome, hypertrophy, atrophy, skeletal muscle, AICAR, mTOR, protein synthesis

## Abstract

AMPK (5’-adenosine monophosphate-activated protein kinase) is heavily involved in skeletal muscle metabolic control through its regulation of many downstream targets. Because of their effects on anabolic and catabolic cellular processes, AMPK plays an important role in the control of skeletal muscle development and growth. In this review, the effects of AMPK signaling, and those of its upstream activator, liver kinase B1 (LKB1), on skeletal muscle growth and atrophy are reviewed. The effect of AMPK activity on satellite cell-mediated muscle growth and regeneration after injury is also reviewed. Together, the current data indicate that AMPK does play an important role in regulating muscle mass and regeneration, with AMPKα1 playing a prominent role in stimulating anabolism and in regulating satellite cell dynamics during regeneration, and AMPKα2 playing a potentially more important role in regulating muscle degradation during atrophy.

## 1. Introduction

5’-adenosine monophosphate-activated protein kinase (AMPK) is an intracellular sensor of ATP consumption that emerged in the late 1990s as a key regulator of skeletal muscle metabolism [1,2,3]. Its role in the promotion of ATP-producing catabolic processes involved in glucose and fat oxidation is well characterized. Its general identity as a catabolic agent is further illustrated by its stimulation of protein degradation and autophagy [4,5,6]. Additionally, AMPK inhibits anabolic processes that consume ATP, such as protein synthesis [7]. Given these general actions, AMPK’s potential negative effect on skeletal muscle growth has been well-studied over the past 20 years.

In this review, a very brief overview of AMPK structure and function will be presented. Then, AMPK’s effect on cell processes that are relevant to the control of cell size, such as protein synthesis, protein degradation and autophagy, will be reviewed. Finally, the known experimental effects of AMPK modulation on skeletal muscle growth and regeneration will be presented.

## 2. AMPK and Its Activation

### 2.1. AMPK Structure and Activation

Many excellent sources are available in the literature that provide a thorough review of the molecular and mechanistic details of AMPK structure and activity (e.g., [3,8,9]). Only a brief summary is provided here. Active AMPK is a heterotrimer comprised of three subunits: α, β, and γ. The actual kinase domain is contained within the α subunit, along with the predominant regulatory phosphorylation site, Thr172, which must be phosphorylated to produce any significant activity. The α and γ subunits serve scaffolding and regulatory roles. The γ subunit confers AMP sensitivity to the enzyme through four cystathionine β-synthase (CBS) domains, which can bind AMP, ADP, or ATP. This interaction with three of these nucleotides confers on AMPK its ability for effectively detecting cellular energy status. During energy stress, when ATP breakdown to ADP accelerates, AMP is generated through the action of adenylate kinase, which transfers a phosphate from one ADP molecule to another, resulting in the production of ATP and AMP. As AMP levels rise, it (and to some degree, ADP) activates AMPK by: (1) increasing AMPK phosphorylation by upstream kinases; (2) decreasing AMPK dephosphorylation by phosphatases; and (3) allosterically activating phosphorylated AMPK [8]. AMPK’s response to the decrease in the ATP:AMP ratio are crucial for the cell’s ability to maintain appropriate ATP levels because it promotes ATP-generating catabolic processes, while inhibiting ATP-consuming anabolic processes [3,8,9].

Different isoforms exist for each of the AMPK subunits. Two α (α1 and α2), two β (β1 and β2), and three γ (γ1, γ2, and γ3) isoforms result in the possibility of up to 12 distinct AMPK configurations. In human skeletal muscle, however, these configurations are likely limited to α2/β2/γ1 (most abundant), α2/β2/γ3, and α1/β2/γ1 [10]. Of these three, α2/β2/**γ**3 accounts for the majority of AMPK activation due to high-intensity exercise [11]. In contrast to human muscle, mouse muscle contains β1 trimers (α1β1**γ**1 and α2β1γ1), although these still only contribute slightly to the overall AMPK activity [12]. While some functional implications of these different configurations have been determined, a full understanding of the full impact of differing trimer contents in tissues and within muscles is still being worked out.

### 2.2. Upstream AMPK Kinases

In mature skeletal muscle, liver kinase B1 (LKB1) is generally considered the primary AMPK kinase since total AMPK activity is essentially eliminated by muscle-specific LKB1 knockout [13,14,15,16]. LKB1 seems, however, to play a more important role in AMPKα2 activation, since AMPKα1 activity is not heavily impacted in skeletal muscle by LKB1 knockout [13,14,17,18,19].

Calcium/calmodulin-dependent protein kinase (CamKK) [20,21], and transforming growth factor β-activated kinase-1 (TAK1) [19,22] likely also play important roles in the activation of AMPK in skeletal muscle under certain circumstances.

### 2.3. AMPK Activators in Skeletal Muscle

#### 2.3.1. Exercise

As would be expected given its role as a cellular energy sensor, AMPK is strongly activated in skeletal muscle by repeated muscle contraction [23] and exercise [2,11,24] in both rodents and humans. Activation of AMPKα2-containing trimers by endurance exercise occurs within 5 min of the onset of exercise [25], and likely requires a relatively high intensity effort, usually somewhere above 50% of VO2max [24,26]. AMPK activity returns to baseline levels within 3 h after exercise [26].

While AMPKα2 activity is readily increased by exercise and muscle contraction in rodents [27], increases in AMPKα1 activity after exercise/contraction are less consistent. For example, AMPKα1 activity in mouse quadriceps muscle was approximately four times higher immediately after 90 min of treadmill running at 13–17 m/min [28], but was not activated at all after running at 10–15 m/min for 60 min [29]. Similarly, 30 min of treadmill running at 30% of maximum running capacity activated AMPKα2 in mouse skeletal muscle, but not AMPKα1, while running at 70% of maximum activated both isoforms [30]. In vitro contraction of the extensor digitorum longus (EDL) muscle for 25 min activated AMPKα1, while 20 min of in situ contraction of the tibialis anterior (TA) failed to do so [29]. The data from rodents is confirmed in human studies where cycling for 1 h at 50% and 70% VO2max failed to activate AMPKα1 [24], while a single 30 s sprint [31] or high intensity interval cycling (4 × 30 s bouts of cycle sprints) [32] activated both AMPKα1 and α2 isoforms. Thus, activation of AMPKα1 isoforms by exercise requires greater intensity work and/or duration than for the activation of AMPKα2. As discussed below, this has important implications in relation to AMPK’s impact on muscle growth and repair, as AMPKα1 appears to be critical in the regulation of anabolism.

#### 2.3.2. AICAR

The 5-amino-4-imidazolecarboxamide ribonucleoside (AICAR) has been used for nearly 25 years to activate AMPK in various tissues in the body [33], including skeletal muscle [1,34,35]. Upon administration, it is converted into ZMP (AICAR monophosphate), an AMP mimetic that activates AMPK without altering intracellular adenine nucleotide levels. Similar to relatively low-intensity exercise, intraperitoneal injection of AICAR activates AMPKα2 but not AMPKα1 in rat gastrocnemius [7]. Furthermore, AICAR-stimulated glucose uptake is eliminated in AMPKα2 knockout muscle, but not in AMPKα1 knockouts [36], suggesting that at least some of AICAR’s metabolic effects are specifically AMPKα2 dependent. Nonetheless, AICAR can activate AMPKα1, since incubation of isolated rat epitrochlearis muscle with 2 mM AICAR activated AMPKα1, albeit to a lesser degree than AMPKα2 [27].

#### 2.3.3. Metformin

Metformin has long been used as a front-line drug in the treatment of insulin resistance and diabetes because of its ability to improve hyperglycemia in an insulin-independent manner. Shortly after AMPK’s metabolic actions began to be described, which are similar to those of metformin, it was discovered that at least some of metformin’s effects are, indeed, AMPK-dependent, although some are not [37,38]. The activation of AMPK by metformin is mainly indirect, where metformin inhibits mitochondrial oxidative phosphorylation, thereby decreasing ATP production and generating an energetic stress on the cell [38]. Although the liver is considered the principal site of metformin’s glucose-regulating effects, chronic, therapeutic dosing of metformin over a 10 week period does increase AMPKα2 (but not AMPKα1) activity in diabetic skeletal muscle [39]. However, it isn’t known whether this is a direct effect of the metformin on skeletal muscle since the effect persisted after metformin withdrawal. In mice, a metformin injection modestly increased AMPKα1 and α2 activity, while treatment of isolated epitrochlearis and soleus (SOL) muscle ex vivo with 10 mM (but not 2 mM) metformin markedly activated AMPKα1 and α2 isoforms [40]. However, the relevance of these high concentrations to in vivo metformin action is questionable.

#### 2.3.4. Small Molecule AMPK Activators

A-769662 was the first small molecule AMPK activator described in the literature [41]. It specifically targets β1-containing AMPK trimers, and in skeletal muscle only activates the scarcely-expressed α1β1 complexes [42]. Several additional activators have subsequently been identified, with varying specificities for the different AMPK subunit isoforms. Of them, Ex229 (small molecule 991), PF-739, and MK-8722 have been demonstrated to activate AMPK in skeletal muscle [43], though effects on muscle growth, atrophy, and regeneration are unknown.

## 3. Regulation of Growth-Related Cell Processes by AMPK

Skeletal muscle growth, in essence, occurs when the rate of protein anabolism exceeds the rate of protein catabolism. Atrophy results when protein catabolism exceeds anabolism [44]. AMPK is known to regulate both processes.

### 3.1. Effect of AMPK on Protein Synthesis

The first indications that AMPK played a role in the regulation of protein metabolism came in 2002 when it was shown that the fractional rate of protein synthesis in skeletal muscle deceased approximately 45% 1 h after an injection of the AMPK-activating drug, AICAR [7]. This inhibitory effect of AMPK activation on protein synthesis was subsequently observed in cultured muscle cells [45] as well as hepatocytes/liver [46,47,48], cardiac myocytes [49,50], and cancer cells [51,52], among other cell types and tissues.

AMPK’s inhibition of protein synthesis is mediated by regulation of protein translation through the mechanistic target of rapamycin, complex 1 (mTORC1) pathway. Regulation of mTORC1 activity is complex, as it serves as a signaling checkpoint for many environmental inputs including nutrients, energy status and mechanical strain. When activated, mTORC1 drives cell growth in part by stimulating protein synthesis through its phosphorylation of several downstream targets, the best characterized of which are the 70-kDa ribosomal protein S6 kinase (p70S6K1) and eukaryotic initiation factor 4E-binding protein 1 (4E-BP1).

AMPK has been shown to inhibit mTORC1 activity through multiple mechanisms. First, AMPK phosphorylates mTOR, a key component of the mTORC1 complex, at Thr2446 [53], which is thought to impair mTORC1 activity by preventing phosphorylation at Ser2448. This site (Ser2448) was initially thought to promote mTORC1 activity when phosphorylated. Since then, its relevance to mTORC1 activity has been reassessed, and it seems probable that phosphorylation of both sites (i.e., Thr2446 and Ser2448) is inhibitory on mTORC1 activity [54]. Nonetheless, AMPK also inhibits mTORC1 by phosphorylating tuberous sclerosis complex 2 (TSC2). Activation of mTORC1 occurs at the lysosomal membrane through interaction with GTP-bound Rheb [55]. TSC2 acts, in complex with binding partners tuberous sclerosis complex 1 and TBC1 domain family member 7 (TBC1D7) [56], as a GTPase activating protein that converts GTP to GDP, thereby greatly diminishing the ability of Rheb to promote mTOR activity. Finally, AMPK phosphorylates raptor, an mTOR binding partner that is essential for mTORC1 activity. This phosphorylation leads to sequestration of raptor by 14-3-3 proteins, and impaired mTORC1 activity [57].

In addition to its inhibitory action on mTORC1, AMPK also regulates protein synthesis through inhibition of eukaryotic elongation factor 2 (eEF2) activity. Phosphorylation of eEF2 at Thr56 inhibits binding of the elongation factor to the ribosome, thereby slowing elongation rate. Phosphorylation of eEF2 at this site is mediated by eEF2 kinase (eEF2K). AMPK impacts eEF2K activity in two ways. First, p70S6k phosphorylates and inhibits eEF2K (leading to eEF2 activation), and AMPK can prevent this by inhibiting the mTOR pathway, as described above. Secondly, AMPK directly phosphorylates and activates eEF2K, leading to eEF2 inactivation [47,58]. While translation initiation is often considered the rate-limiting step in protein synthesis, control of elongation can, under certain circumstances, be critical in protein synthetic rate [58,59]. For instance, inhibition of eEF2K partially blocks the acute inhibitory effect of contractions on protein synthesis, although this effect does not appear to be regulated by AMPK [60]. Thus, the capacity for eEF2 regulation by AMPK in skeletal muscle remains unclear.

### 3.2. Effect of AMPK on Catabolic Processes

#### 3.2.1. AMPK and Autophagy

Defective cellular content (organelles, pathogens, etc.) is degraded and recycled through the process of autophagy under low-energy conditions such as nutrient deprivation and exercise. Autophagy involves several subprocesses including engulfment of the target components in an autophagosome, fusion of the autophagasome with a lysosome (forming an autophagolysosome), followed by degradation of the cargo. This is a complex process and a complete description will not be presented here (see reference [61] for an excellent review). However, several key points of autophagy regulation are important in the context of the current topic. Under low-energy conditions, uncoordinated 51-like kinase 1 (ULK1) phosphorylates and activates multiple downstream targets that promote the progression of autophagy, including several autophagy related (ATG) proteins and beclin-1. Under conditions of energy abundance mTORC1 inhibits ULK1 through phosphorylation at Ser757. This, along with its targeting of other autophagy components, leads to mTOR’s inhibition of autophagy [61].

AMPK has long been known to regulate autophagy. Initial observations in rat hepatocytes suggested that AICAR-induced AMPK activation inhibited autophagy [62], but subsequent work demonstrated that the AMPK inhibitor, Compound C, and dominant negative AMPK expression also inhibited autophagy, suggesting that AICAR’s effects might be AMPK-independent [63]. Since then, AMPK’s role in the process remains complicated because its effect seems to be dependent on cell type and metabolic context. Nonetheless, it appears that AMPK generally supports and promotes autophagy [64,65], and this is true in skeletal muscle [66]. It does this through multiple mechanisms. As noted above, AMPK inhibits mTORC1 activity. This relieves mTORC1 inhibition of ULK1, and thereby promotes autophagic flux. Additionally, AMPK directly phosphorylates components of the autophagy regulatory machinery. AMPK phosphorylates ULK1 at several sites [67], and also targets autophagy related protein 9 (ATG9) [4] and beclin-1 [68] downstream of ULK1, promoting autophagy.

#### 3.2.2. AMPK and Ubiquitin-Proteasome Mediated Catabolism

The 26S proteasome degrades proteins that have been tagged for destruction through the attachment of ubiquitin chains. The covalent attachment of ubiquitin to targeted proteins is catalyzed through the action of three enzymes (E1, E2, and E3). E3 actually refers to one of multiple ubiquitin ligases, each of which is specific for the degradation of particular proteins. In skeletal muscle, two E3 enzymes, Atrogin-1 and muscle ring finger-1 (MuRF-1), are known to play a prominent role in proteasomal protein breakdown during muscle atrophy [69].

Expression of the atrophy-related genes Atrogin-1 and MuRF-1 is regulated through members of the forkhead box (FoxO) transcription factors. Anabolic signaling that activates Akt (e.g., via nutritional and hormonal cues mediated by insulin and other growth factors) results in their cytoplasmic localization and subsequent degradation so that they do not induce atrogene transcription. Catabolic stimuli, such as oxidative stress and inflammation, increase MuRF-1 and Atrogin-1 expression in muscle through the mitogen-activated protein kinase (MAPK) p38 as well as nuclear factor-κB (NF-κB) [69].

AMPK stimulates FoxO activity. AICAR injection into mice increases FoxO1 and FoxO3 expression [28,70], although AICAR’s upregulation of FoxO1 is not impacted by knockout of AMPKα2 [28]. Treatment of C2C12 myotubes with AICAR results in protein breakdown accompanied by increased expression of FOXO, Atrogin-1, MuRF-1, and two other FoxO target genes, microtubule-associated protein 1A/1B-light chain 3 (LC3), and Bnip3 [71], and these effects are Akt/mTOR independent [6]. AMPK also phosphorylates FoxO3a at a site known to activate the transcription factor and thereby induce generalized protein degradation but this may not necessarily affect its localization in the nucleus [72,73,74]. AICAR also increases Foxo3 binding to the MuRF-1 and Atrogin-1 promoters [6]. Furthermore, AMPK activation increases nicotinamide adenine dinucleotide (NAD^+^) concentration which activates the sirtuin 1 (SIRT1) deacetylase. SIRT1-mediated deacetylation of FoxO proteins increases their transcriptional activity [75,76].

## 4. Influence of AMPK on Skeletal Muscle Size

### 4.1. AMPK Regulation of Basal Muscle Size

Initial observations in dominant-negative AMPK (AMPK-DN) transgenic mice in which a dominant negative AMPKα2 subunit was overexpressed under the muscle creatine kinase promoter (expressed in heart and skeletal muscle) showed that EDL muscles tended to be larger than in wild-type (WT) mice, suggesting that AMPK might negatively regulate basal muscle mass [77], as would be expected given AMPK’s stimulation of catabolism and activation of anabolism. Those initial findings are consistent with later work in which skeletal muscle specific AMPKα1 and α2 double knockout (AMPKα1/α2 dKO) soleus muscles were larger by mass and fiber diameter compared to WT muscles [78]. Myotubes derived via primary muscle cell cultures from these AMPKα1/α2 dKO muscles were likewise larger than from those from WT muscles [78]. On the other hand, muscle-specific AMPKβ1/β2 double knockout (AMPKβ1/β2 dKO) SOL and EDL muscles were reportedly not different in size compared to WT muscles [79]. Why the double knockout of β isoforms did not lead to increased muscle size is not clear, but might be related to the promoter used to drive the Cre-mediated deletion of floxed AMPK. In the case of the AMPKβ1/β2 dKO mice, the muscle creatine kinase (MCK; cardiac and skeletal muscle specific) promoter was used, while in the AMPKα1/α2 dKO mice it was the human skeletal muscle actin (HSA; skeletal muscle specific) promoter. Alteration of AMPK activity in the heart in the AMPKβ1/β2 dKO mice could have influenced the response of skeletal muscle to the knockout since cardiac dysfunction is well-known to induce skeletal muscle atrophy. Thus, not all AMPK deficiency models support the notion that AMPK inhibits basal muscle mass, but these findings are usually derived from AMPK knockout models that are not specific to skeletal muscle. Plantaris muscles from germline AMPKα1 knockout mice are smaller than those from WT mice [80]. The lack of AMPKα1 in all tissues in this model, however, doesn’t allow for conclusions regarding the role of AMPK in the muscle specifically, since the lack of AMPKα1 in other tissues may have impacted muscle size (e.g., by decreasing systemic growth factors or other humoral inputs). Furthermore, the lack of AMPKα1 was associated with compensatorily elevated AMPKα2 activity in the muscles, which could have resulted in decreased mass. In agreement with this interpretation, primary cultured myotubes derived from these cells (removed from the systemic environment of the mouse) were larger than WT muscles [80]. Another study found that muscle fibers, especially type IIb fibers, are smaller in whole-body AMPKβ2 knockout mice [81]. The TA muscles from the aforementioned cardiac/skeletal muscle AMPK β1/β2 dKO mice are also smaller vs. WT muscles [82]. Again, the smaller size of these muscle fibers in these studies could be secondary to systemic effects of altered function of other tissues (e.g., heart), though this has not been directly tested.

### 4.2. Role of AMPK in Skeletal Muscle Hypertrophy

Given AMPK’s pro-catabolic and anti-anabolic actions, it was hypothesized that AMPK activity would block overload-induced muscle growth, and the available data generally support this. When comparing the hypertrophic response of rat muscle to synergist ablation-induced overload, AMPK phosphorylation in the hypertrophying muscle was associated with decreased muscle hypertrophy [83], and diminished mTOR pathway signaling [84]. Several subsequent studies have reported negative associations between AMPK phosphorylation or activity and skeletal muscle growth. Indeed, impaired overload hypertrophy in obese rats [85,86], attenuated mTOR phosphorylation in metabolic syndrome patients [87], myotube hypertrophy during differentiation [88], myostatin inhibition of eEF2 and protein synthesis in myotubes [89], and differences in hypertrophy with varied ladder-climbing protocols in rats [90] are all associated inversely with AMPK activity.

Direct pharmacological evidence showing that AMPK inhibits muscle growth has also been demonstrated. An AICAR injection 1 h prior to a bout of resistance exercise-mimicking contractions greatly attenuated the mTOR signaling response to the contraction bout [35], suggesting that AMPK activation would impair the normal increase in protein translation that occurs post resistance exercise. Likewise, continuous perfusion of overloaded plantaris muscles with AICAR after synergist ablation greatly attenuated muscle hypertrophy [91].

Genetic evidence for the inhibitory effect of AMPK on in vivo skeletal muscle hypertrophy was provided by Mounier, et al. [80], who performed synergist ablations on AMPKα1 knockout mice. After 7–21 days of overload, AMPKα1 expression and activity was significantly increased in WT mice (but not AMPKα1-KO mice, as expected). Despite lower basal muscle mass, whole muscle hypertrophy and muscle fiber hypertrophy at 7 and 21 days was greater for the AMPKα1 knockouts. In line with the hypertrophy measurements, mTOR pathway signaling, as assessed by p70S6k and 4E-BP1 phosphorylation was greater, while eEF2 phosphorylation was lower (corresponding to increased eEF2 activity) after overload in the AMPKα1-KO muscles. Importantly, this occurred despite a compensatory increase in AMPKα2 activity basally and at 7 and 21 days after overload in the KO muscles, demonstrating that AMPKα1 is likely the major isoform involved in regulation of overload-induced muscle growth.

On a related note, old age leads to the loss of muscle mass (sarcopenia) and a blunted anabolic response to hypertrophic stimuli. AMPKα2 activation by exercise and AICAR is typically blunted in old age [34,92]. However, AMPK phosphorylation in old overloaded muscle is elevated vs. young overloaded muscles and is negatively correlated to mTOR signaling and hypertrophy [83,84]. Similarly, AMPK phosphorylation 1–3 h after resistance exercise is elevated in old vs. young human muscle, and is associated with delayed mTOR pathway activation [93]. Interestingly, 10 min of continuous electrically stimulated muscle contractions resulted in increased AMPKα2 activity in muscles from young adult (8 months-old) and old (30 months-old) rats, but this response was attenuated by old age [34]. However, under this stimulation protocol, AMPKα1 activity increased after stimulation only in old muscle, and not young, suggesting that old muscle may be hypersensitive to exercise-induced AMPKα1 activation which perhaps contributes to sarcopenia.

### 4.3. Role of AMPK in the Regulation of Skeletal Muscle Atrophy

The response of the AMPK system to muscle atrophy is unclear. Disuse atrophy of rodent skeletal muscle after 1–4 weeks of hindlimb unloading (HU) has been reported to increase [94] or decrease AMPK phosphorylation [95,96,97], while others observed no effect of HU on AMPKα1 and AMPKα2 activity or on acetyl-CoA carboxylase (ACC; AMPK target and marker of AMPK activity) phosphorylation [98]. Similarly, AMPK activity is reported by some to increase after 4 and 7 days of denervation in mice and/or rats [99,100,101,102], while spinal cord transection does not alter AMPKα2 activity in muscle [103]. In the denervation model, these conflicting results may be due to timing since AMPK phosphorylation in denervated soleus muscles is decreased during at least the first 24 h post-denervation, is not different at 3 days, and is elevated by 7 days post-denervation [104] compared to control muscles. Thus, it appears that AMPK activity in general decreases initially, then increases later on during the adaptation to disuse, at least in the denervation model.

Consistent with a catabolic role for AMPK, HU-induced atrophy of the soleus muscle was partially attenuated in AMPK-DN soleus muscles, potentially through decreased ubiquitin-proteosome activity [98]. It should be noted, however, that in this model, AMPKα1 activity was only mildly decreased by dominant-negative (DN) expression in the transgenic muscles, so the anti-atrophy effect was mainly due to the loss of AMPKα2 activity. Similarly, atrophy in denervated TA muscles from AMPKα2-KO mice was partially blocked compared to WT muscles [101], and this was also associated with decreased autophagic markers, Atrogin-1/MuRF-1 expression and ubiquitination. Akt and 4E-BP1 phosphorylation were unaffected by AMPKα2-KO, suggesting that the attenuation of atrophy was due to decreased protein degradation rather than increased mTOR activity and synthesis. Together, these findings suggest that in contrast to AMPKα1’s role in inhibiting skeletal muscle mTOR and hypertrophy, the presence of AMPKα2 plays a more pronounced role in supporting an atrophy response to disuse, and in promoting protein degradation through the ubiquitin-proteasome system.

While the lack of AMPKα2 attenuates atrophy, increased activation of AMPK above normal does not appear to accelerate the loss of muscle mass since daily AICAR injections during 3 days of tibial nerve denervation in rats did not significantly affect skeletal muscle atrophy in soleus and gastrocnemius muscles [105]. Furthermore, 4 weeks of AICAR treatment of mdx mice (a model for Duchenne muscular dystrophy) did not exacerbate atrophy associated with dystrophy, and actually improved muscle function, probably through enhanced autophagic clearing of damaged cell components [106], and/or promotion of a more oxidative muscle phenotype [107].

### 4.4. Effect of Disruption of LKB1 on Skeletal Muscle Size and Hypertrophy

LKB1 knockout in skeletal muscle results in a nearly complete elimination of basal, exercise, and AICAR-induced AMPKα2 activity [14,18,19] and overall AMPK phosphorylation [13,16,108,109,110], while it has little [13,14] to no [17,18] effect on AMPKα1 activity, which is an important consideration since AMPKα1 seems to be the major isoform regulating muscle growth [78,80]. LKB1 also phosphorylates several other AMPK family members, at least one of which, sucrose non-fermenting 1 AMPK related kinase (SNARK) is important in the maintenance of muscle mass [111].

The weight of muscles from relatively young mLKB1-KO mice is not statistically different from that of WT muscles [16,19]. However, after approximately 30 weeks of age, muscle mass begins to decline in mLKB1-KO muscles [15]. This atrophy is associated with the development of heart failure, however [15,112], and thus may be primarily due to cardiac cachexia. Consistent with that speculation, muscle weights were, for the most part, similar in a skeletal muscle specific dominant negative LKB1 model, though quadricep muscles were smaller, and diaphragms were larger [113].

Nonetheless, since LKB1 is a primary upstream activator of AMPK in skeletal muscle, McGee et al. [19] hypothesized that the lack of LKB1 in muscle would result in a greater hypertrophic response to overload. However, when plantaris muscles from conditional muscle-specific LKB1 knockout mice (mLKB1-KO; cardiac and skeletal muscle knockout) were overloaded via synergist ablation (of the gastrocnemius muscle), there was no significant difference in the degree of mTOR pathway activation or hypertrophy compared to WT muscles. Importantly, overload increased the activity of AMPKα1 (but not α2) in both WT and KO muscles, showing that this response is regulated at least in part in an LKB1-independent fashion, perhaps via CamKK or TAK1 signaling [19]. Therefore, based on the findings of Monier, et al., that it is the α1 subunit that regulates skeletal muscle mTOR signaling and size [80], the lack of difference in hypertrophic response in the mLKB1-KO muscles is not surprising.

However, when skmLKB1-KO muscles were subjected to an acute bout of intermittent contractions designed to mimic hypertrophy-inducing resistance exercise, mTOR signaling (p70S6k and ribosomal protein S6 phosphorylation) was elevated to a greater extent both basally and immediately post-contraction in knockout vs. WT muscles, as was protein synthesis at 8 h post contraction [110]. AMPK phosphorylation was increased with contractions in WT but not skmLKB1-KO muscles using this contraction protocol, suggesting that the increased mTOR signaling in the knockout muscle could be due to a lack of AMPK activation, but AMPKα1-specific activity was not measured. This suggests that LKB1 can exert catabolic effects under some circumstances, though evidence that this impacts gross muscle hypertrophy is lacking.

Potential effects of LKB1 on muscle atrophy during unloading or denervation are currently unknown.

### 4.5. Exercise-Induced AMPK Activation and Muscle Hypertrophy

That endurance training interferes with hypertrophy/strength gains has been well-established [114,115]. The accumulation of evidence demonstrating AMPK’s anti-anabolic and pro-catabolic effects naturally leads to the question of whether its activation during exercise functionally impairs the ability of muscle to hypertrophy, which, if true, would mechanistically explain the conflict between endurance/hypertrophy responses. In support of this hypothesis, Atherton et al. [116] showed that tissue-autonomous differences in signaling pathway activation may contribute to the inherent differences in gross adaptation that is observed with endurance vs. resistance exercise training. Using in vitro electric stimulation protocols that mimic endurance (low frequency, continuous) and resistance (high-frequency, intermittent) exercise bouts in rat skeletal muscle, they showed that endurance-type stimulation (but not resistance-type stimulation) resulted in AMPK activation and accrual of peroxisome-proliferator-activated receptor γ coactivator-1 α, while resistance-type stimulation (but not endurance-type stimulation) increased phosphorylation of Akt, TSC2, mTOR, downstream mTOR targets, and increased protein synthesis.

In humans, however, the molecular responses to different exercise modalities is less clear and has generally been interpreted as not supporting the hypothesis that physiological AMPK activation (e.g., through endurance exercise training) significantly impacts mTOR signaling and/or protein synthesis [115]. Apró et al. showed in trained male subjects that activation AMPKα2 via 1 h of intense cycling did not significantly impair subsequent activation of mTOR pathway components or mixed muscle fractional protein synthesis after a resistance training bout [117]. However, AMPKα1 was not activated by either exercise bout in this case. Since AMPKα1 is the major AMPK isoform regulating skeletal muscle growth, at least in rodents [80], the lack of an effect of this endurance exercise bout on mTOR or protein synthesis would be expected and does not preclude an AMPK effect if the α1 subunit were actually activated (e.g., by more intense or prolonged exercise than that employed in this study).

Furthermore, while acute AMPK activation immediately after exercise is suppressed in endurance-trained muscle [118,119], chronic endurance exercise training increases basal AMPKα1 protein content and activity. Twelve weeks of treadmill training (90 min/day, 5 days/week) elevated both AMPKα1 and α2 protein content in rat muscle [120]. Similarly, in humans, AMPKα1 (but not AMPKα2) protein concentration [118,121,122] and basal AMPKα1 activity [122] is greater in endurance trained vs. untrained individuals. Thus, the question of whether or not resistance exercise-induced anabolic signaling and hypertrophy are impacted by AMPKα1 activation by endurance exercise training remains unresolved.

### 4.6. Does Pharmacological AMPK Activation Limit Skeletal Muscle Hypertrophy?

Data showing the effect of pharmacological AMPK activation on load-induced muscle hypertrophy is quite limited. As noted previously, AICAR activation of AMPK attenuates contraction-induced increases in mTOR signaling and overload-induced hypertrophy in rodent muscles [35,91].

Interestingly, metformin treatment of patients with severe burn injury at dosages (850–2550 mg/day for 8 days) previously shown to activate AMPKα2 but not AMPKα1 in skeletal muscle [39] led to a significant increase in protein synthesis [123]. Similarly, in tumor-bearing cachexic rat muscle, metformin treatment rescued protein synthesis and decreased protein degradation while activating AMPK, though isoform-specific activity measures were not taken [124]. This improvement in muscle anabolism may be attributable to the long-appreciated impact of metformin on insulin sensitivity. Improved insulin action at the skeletal muscle would not only improve glucose handling, but protein synthesis as well.

## 5. Influence of AMPK on Skeletal Muscle Regeneration after Injury

### 5.1. The Regenerative Process in Skeletal Muscle

Skeletal muscle regeneration after injury is dependent upon the action of muscle stem cells (MuSCs), primarily satellite cells (SCs) which, in uninjured muscle, reside underneath the basal lamina next to mature muscle fibers in a quiescent, mitotically inactive state. Upon muscle damage, these cells activate and proliferate, with their subsequent progeny either engaging in a process of self-renewal to maintain the MuSC pool, or differentiating into myoblasts that then fuse together with other myoblasts or existing myofibers, leading to repair or replacement of the damaged tissue. Many excellent reviews are available for more detail on these events (e.g., [125,126,127]).

Muscle regeneration is a precisely ordered process that is dependent on the actions and influence of many cellular players at or near the myogenic niche, including SCs, mature muscle fibers, immune cells, fibroblasts, fibroadipogenic progenitors (FAPs), and others [125,126]. Although AMPK likely plays an important role in the regulation of many of these cell types (in macrophages, for instance [128]), the discussion here will be limited to its role in SCs.

### 5.2. Effect of AMPK on Myogenesis in Culture

The C2C12 adult skeletal muscle myoblast cell line is frequently used as an in vitro culture model for studying the process of myogenesis. C2C12 myoblasts, prior to differentiation in low-serum media, express the α2, γ2, and γ3 AMPK isoforms, but minimal expression of α1, β1, β2, and **γ**1 isoforms. Differentiation of the myoblasts into myotubes by exposure to low-serum media, however, induces the expression of all isoforms except for γ1 [129]. Consistent with the lack of β isoforms in myoblasts, which should preclude AMPK activation, stimulation of the cells with oligomycin and serum withdrawal activated AMPK much more strongly in myotubes vs. myoblasts [129]. However, other findings show that AICAR is able to activate AMPK in undifferentiated myoblasts [130], suggesting that the lack of β isoforms and AMPK activity in myoblasts is not a generalizable finding.

Activation of AMPK impairs myoblast proliferation. When C2C12 myoblasts are cultured in low glucose conditions (≤5 mM), AMPK is activated leading to impaired differentiation into myotubes. The same phenomenon is true for primary myoblasts, but only at even lower glucose concentrations [131]. Pharmacological AMPK activation with AICAR, metformin and other drugs accomplishes the same impairment in differentiation [130,131].

AMPK also impairs myoblast differentiation in culture. Activation of AMPK with AICAR in differentiating C2C12 myoblasts decreased p21 expression (which normally increases dramatically during differentiation) and cell cycle transition, and decreased myotube formation and myosin heavy chain expression [130]. A similar inhibitory effect of AICAR on primary bovine myoblasts was also observed [132]. Furthermore, transfection of C2C12 myoblasts with CamKKβ, an established AMPK activator, resulted in AMPK activation in myoblasts, cell cycle arrest and impaired proliferation as well as impaired subsequent differentiation, and this effect on proliferation and differentiation was AMPK-dependent since it was blocked by dominant-negative AMPK expression [133]. Together, these in vitro findings suggest that hyperactivation of AMPK in myoblasts blocks muscle proliferation and differentiation.

### 5.3. Effect of AMPK on Muscle Regeneration In Vivo

Although hyperactivation of AMPK in culture impairs both proliferation and differentiation of myoblasts, the lack of AMPK in SCs in vivo blocks normal muscle regeneration after injury. AMPKα1 is the predominant catalytic isoform in quiescent, activated and differentiating satellite cells [134,135,136]. Regeneration of damaged muscle is impaired (vs. WT) in both constitutive AMPKα1-KO mice, as well as in mice with AMPKα1-KO induced just before injury [137], and this is associated with decreased satellite cell number and Pax7, myogenic factor 5 (Myf5), and myogenin expression in basal muscles. Furthermore, AMPKα1-KO satellite cells have diminished myogenic capacity when transplanted into WT muscles, showing that the defect in regeneration is mediated by the lack of AMPKα1 in the satellite cells themselves, rather than in other cells in the KO animals (such as fibroblasts, macrophages, etc.) [137].

A similar impairment of regeneration is demonstrated by satellite cell-specific AMPKα1-KO. Fu et al. reported that satellite cells lacking AMPKα1 activate and proliferate more slowly both in culture and in single fiber preparations, and result in a subsequent impairment of muscle regeneration after cardiotoxin injury [136]. SCs, with their scant mitochondria, depend heavily on glycolytic metabolism and, according to the findings of these authors, the lack of AMPK impairs SC activation and proliferation by decreasing Warburg-like glycolysis [136].

Theret et al. also reported that satellite cell-specific AMPKα1-KO impairs muscle regeneration [135]. They showed that when SCs from AMPKα1 knockout mice (but not AMPKα2 knockouts) were collected and differentiated, the lack of AMPKα1 resulted in increased self-renewal instead of differentiation [135]. Similarly, deletion of AMPKα1 in MuSCs in vivo resulted in decreased size of the regenerating fibers along with decreased differentiation and fusion, but increased proliferation of MuSCs. However, in contrast to the report of Fu et al., the impaired regeneration was attributed by these authors to increased lactate dehydrogenase activity and enhanced Warburg-like glycolysis in the AMPKα1-KO SCs. The reason for this discrepancy is not clear, but could be due to different transgenic constructs. Regardless, both studies demonstrate the importance of SC AMPKα1 in allowing for proper regeneration through metabolic regulation. Together with the culture data, the available evidence indicates that AMPK activity must be kept within relatively tight bounds (not too high or too low) for optimal muscle regeneration.

### 5.4. LKB1’s Role in Skeletal Muscle Regeneration

The content of the upstream AMPK kinase, LKB1, increases during myoblast differentiation [138]. Overexpression of LKB1 in C2C12 myoblasts enhances differentiation, while RNAi-mediated knockdown of LKB1 impairs differentiation [138]. While some of this effect is likely due to the action of LKB1 on other targets within the AMPK family, AMPK phosphorylation is also increased substantially during muscle differentiation [138].

The lack of LKB1 in SCs promotes proliferation and self-renewal of the satellite cell pool, but impairs myoblast differentiation [139,140]. The effect on self-renewal is due in part to the activation of the Notch signaling pathway in LKB1-deficient cells, leading to overexpression of Pax7 that appears to be dependent on the decreased AMPK activation in these cells [139]. Other findings indicate that AMPKα1 also regulates self-renewal in a LKB1-independent manner [135]. Furthermore, LKB1’s role in SC differentiation is at least partly independent of AMPK through regulation of glycogen synthase kinase (GSK3)/Wnt signaling [140].

## 6. Conclusions and Future Perspectives

**Figure 1:**
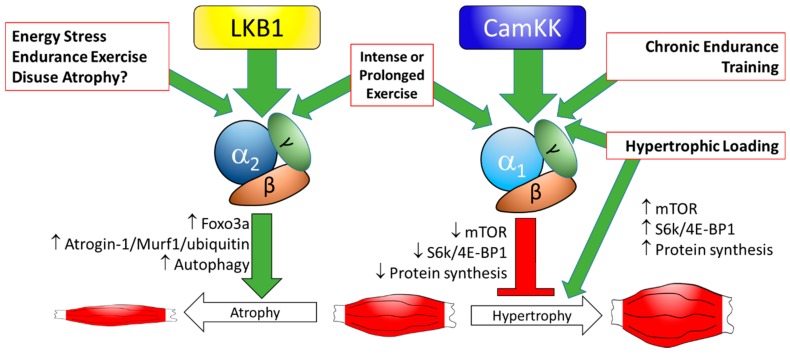
Proposed regulation of skeletal muscle size by 5’-adenosine monophosphate-activated protein kinase (AMPK). Energy stress (decreased ATP/AMP ratio; as in moderately intensive exercise) predominantly activates AMPKα2 via liver kinase B1 (LKB1), while AMPKα1 is only activated by highly-intense or prolonged exercise. Basal AMPKα1 content and activity is also increased by long-term endurance training, perhaps via Calcium/calmodulin-dependent protein kinase (CamKK) action, or other AMPK kinases. AMPKα2 stimulates catabolic processes by increasing Foxo3a, Atrogin-1 and MuRF-1 expression/activity and increasing autophagy, leading, under certain circumstance, to muscle atrophy, but has little effect on protein anabolism. AMPKα1 impairs mTOR signaling, slows protein synthesis, and blocks hypertrophy. Hypertrophic loading (i.e., resistance exercise) stimulates mechanistic target of rapamycin (mTOR) signaling, protein synthesis, and hypertrophy, but also activates AMPKα1 independent of LKB1 (perhaps via CamKK or other means), limiting the hypertrophic growth. ↑: increase expression or activity; ↓: decreased expression or activity.

AMPK’s role as a signaling nexus for cellular processes that control energy balance has been well established over recent decades. While it certainly is not the only player in the regulation of skeletal muscle development, size, and/or growth, it, and especially the AMPKα1 subunit, has emerged as a key factor that limits muscle size and capacity for hypertrophy. AMPKα2, on the other hand, may play a more substantial role in promoting muscle atrophy than AMPKα1 through its actions on autophagy and protein degradation (summarized in Figure 1). AMPK also limits myogenesis and regeneration after injury, although the loss of AMPKα1 also blocks these processes, showing that some (but not too much) AMPK activity is required for proper regenerative functioning. While many questions regarding AMPK’s role in muscle growth and regeneration have been answered, others still remain unanswered. Does AMPKα1-specific activity after endurance exercise interfere with concomitant resistance-training adaptations? What cellular mediators control AMPK’s effects on muscle growth and development? How does AMPK activity in neighboring accessory cells support or impair satellite cell function in muscle regeneration? Can pharmacological AMPK activation or inhibition be harnessed to improve hypertrophic and regenerative responses, especially in populations where these are impaired (aging, obesity, diabetes, myopathies, etc.)? What role do LKB1 and other AMPK family members play in these processes? Continuing work in this area will surely shed additional light on these and other important questions.

## Abbreviations

4E-BP1	eukaryotic initiation factor 4E binding protein 1
ACC	acetyl-CoA carboxylase
AICAR	5-amino-4-imidazolecarboxamide ribonucleoside
AMPK	AMP-activated protein kinase
CamKK	calcium/calmodulin-dependent protein kinase
CBS	cystathionine β-synthase
dKO	double knockout
DN	dominant negative
EDL	extensor digitorum longus
eEF2	eukaryotic elongation factor
eEF2K	eEF2 kinase
FAP	fibroadipogenic progenitor
FoxO	forkhead box
GSK3	glycogen synthase kinase 3
HU	hindlimb unloading
HSA	human skeletal muscle actin
KO	knockout
LC3	microtubule-associated protein 1A/1B-light chain 3
LKB1	liver kinase B1
MAPK	mitogen activated protein kinase
MCK	muscle creatine kinase
mLKB1-KO	muscle-specific LKB1 knockout
mTORC1	mechanistic target of rapamycin, complex 1
MuRF-1	muscle ring finger-1
Myf5	myogenic factor 5
NF-κB	nuclear factor kappa B
SC	satellite cell
SIRT-1	sirtuin 1
skmLKB1-KO	skeletal muscle-specific LKB1 knockout
SNARK	sucrose non-fermenting 1 AMPK related kinase
SOL	soleus
TA	tibialis anterior
TAK1	transforming growth factor β-activated protein kinase
TBC1D7	TBC1 domain family member 7
TGFβ	transforming growth factor β
TSC2	tuberous sclerosis complex 2
ULK1	uncoordinated 51-like kinase 1
Wnt	wingless/integrated
WT	wild-type
